# Expression and prognosis analysis of *TBX2* subfamily in human lung carcinoma

**DOI:** 10.1007/s12672-024-00900-w

**Published:** 2024-02-27

**Authors:** Rui Mi, Qiubo Wang, Qingyang Liu, Fengying Jiang, Yuan Ji

**Affiliations:** 1 Department of Clinical Laboratory, Wuxi 9Th People’s Hospital Affiliated to Soochow University, No.999 Liang Xi Road, Binhu District, Wuxi, 214000 Jiangsu China; 2 School of Medicine, Soochow University, Suzhou, 215123 Jiangsu People’s Republic of China

**Keywords:** *TBX2* subfamily, Lung carcinoma, Prognosis, Methylation

## Abstract

**Purpose:**

Lung cancer has a high morbidity and mortality rate of all cancers worldwide. Therefore, there is an urgent need for reliable cancer markers for diagnosis and prognosis of patients with lung cancer.

**Methods:**

In this study, we used the bioinformatics database to compare the expression of the *TBX2* subfamily at the transcriptional and protein levels in non-small cell lung cancer. Then, to confirm our bioinformatics analysis above, we used western bloting to determine the expression of *TBX2*, *TBX3*, *TBX4* and *TBX5* in human lung squamous carcinoma cell lines. Besides, low expression of *TBX2* subfamily predicted a poor prognosis of patients with lung cancer. Finally, The methylation database was used to explore the relationship between the low expression of *TBX2* subfamily and methylation of gene promoter region.

**Results:**

Our data showed a significant decrease of *TBX2* subfamily expression in lung cancer tissues of several histological subtypes. Finally, the methylation of *TBX2* subfamily members in the promoter region of NSCLC was significantly higher than that in normal tissues.

**Conclusion:**

Our research provided sufficient evidence that *TBX2* subfamily might play an inhibitory role in malignancy progression of lung cancer, which is promising to shed light on discovering a novel reliable cancer marker for prognosis of lung cancer patients.

## Introduction

Lung cancer has the highest mortality rate of all cancers worldwide [1]. Every year, approximately 156 000 people die from lung cancer, even more than the next three most incident cancers combined. More women have died from lung cancer than breast cancer since 1987 [2]. Although smoking is one of the most important risk factors contributing to lung cancer, the mortality of non-smoking lung cancer patients has already risen to the seventh position [3]. Among several histological subtypes of lung cancer, non-small cell lung cancer (NSCLC) accounts for the majority of new diagnosis at 85%, while small cell carcinoma only accounts for the remaining 15% [4]. The histological subtypes of NSCLC includes: large cell carcinoma, squamous cell carcinoma and adenocarcinoma accounting for 10, 25 and 40% separately [5]. The rest are mainly carcinoid tumors and bronchalveolar carcinoma. Although some clinicopathological parameters including tumor stage, size, and lymph node metastasis have been proven to be reproducible prognostic determinants in lung cancer, they are far from sufficient to explain the individual variability [6]. Therefore, there is an urgent need for reliable cancer markers for diagnosis and prognosis of patients with lung cancer.

T-box family members play important role in regulation of embryonic development, and particularly in morphogenesis and the assignment of cell fate [7]. *TBX2* subfamily is a member of T-box transcription factors, which includes the closely related genes such as *TBX2*, *TBX3*, *TBX4* and *TBX5* [8]. T-box proteins contain a highly conserved T-domain which can recognize the core sequence GGTGTGA (T-element) and affect dimerization and DNA binding [9]. T-box proteins function as putative transcription factors which regulate the expression of downstream targeted genes. Recently, it has been reported that *TBX2* subfamily members might be involved in generation and development of tumors [10]. Recent studies indicated that *TBX2* was amplified in 8.6%-21.6% of sporadic human breast carcinomas, where the protein was overexpressed [11]. Besides, ectopic expression of *TBX2/3* contributed to chemotherapy resistance, DNA polyploidy and tumor proliferation [12]. It was also reported that the overexpression of *TBX2* in prostate cancer was correlated with pathological grade and tumor stage and might act as a potential cancer marker in prostate cancer [13]. On the other hand, low expression of *TBX4* in patients with pancreatic ductal adenocarcinoma (PDAC) predicted a poor prognosis [14]. Taken together, these evidences suggest that *TBX2* subfamily genes may function as tumor promoting or inhibiting factors and play vital role in cancer progression.TBX2 subfamily controls lung growth by repressing the cell cycle inhibitor genes Cdkn1a and Cdkn1b [15]. TBX2 subfamily function downstream of Shh by directly repressing the Wnt antagonists Frzb and Shisa3, thereby maintaining the proliferative mesenchymal Wnt signaling [16]. TBX2 subfamily also maintains the proliferation of lung mesenchyme through at least two molecular mechanisms: regulating cell cycle control and integrating the activity of multiple signaling pathways. However, TBX2 subfamily regulates the proliferation of lung interstitial cells through various mechanisms, yet its regulatory mechanism as a transcription factor in lung cancer remains unclear. Previous studies have reported low expression of the TBX2 subfamily genes in lung cancer tissue. Previous studies have reported low expression of the TBX2 subfamily genes in lung cancer tissue, the relationship between low expression of *TBX2* subfamily and prognosis of lung cancer still remains elusive [17]. Here on this basis, we further sought to survey the relationship between *TBX2* subfamily expression and prognosis of lung cancer by bioinformatics and verified it in human lung cancer specimens. Finally, we used two databases to verify the epigenetic mechanism of low expression of *TBX2* subfamily.

## Results

### The structure of TBX2 subfamily transcripts

**Fig. 1:**
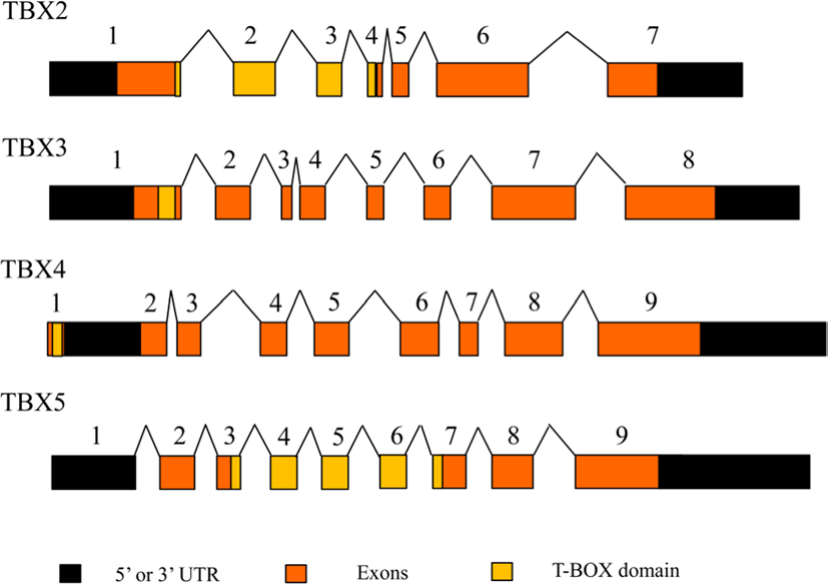
Graphical structure of *TBX2* subfamily transcription products

According to the classification of HUGO gene nomenclature committee (HGNC), there are four members in TBX2 subfamily, including *TBX2*, *TBX3*, *TBX4* and *TBX5*. Based on the data from the HUGO database, we used drawing software to draw the gene structure diagram of the TBX2 subfamily genes. The structure of *TBX2* subfamily genes including the 5’ or 3’-untranslated region, exons region and T-BOX specific domain was displayed in detail in the lower panel of Fig. 1. T-BOX domain is a highly conserved DNA-binding domain consisting of 180 amino acids on the N terminal.

### Transcription levels of TBX2 subfamily in different types of cancers (UALCAN)

**Fig. 2:**
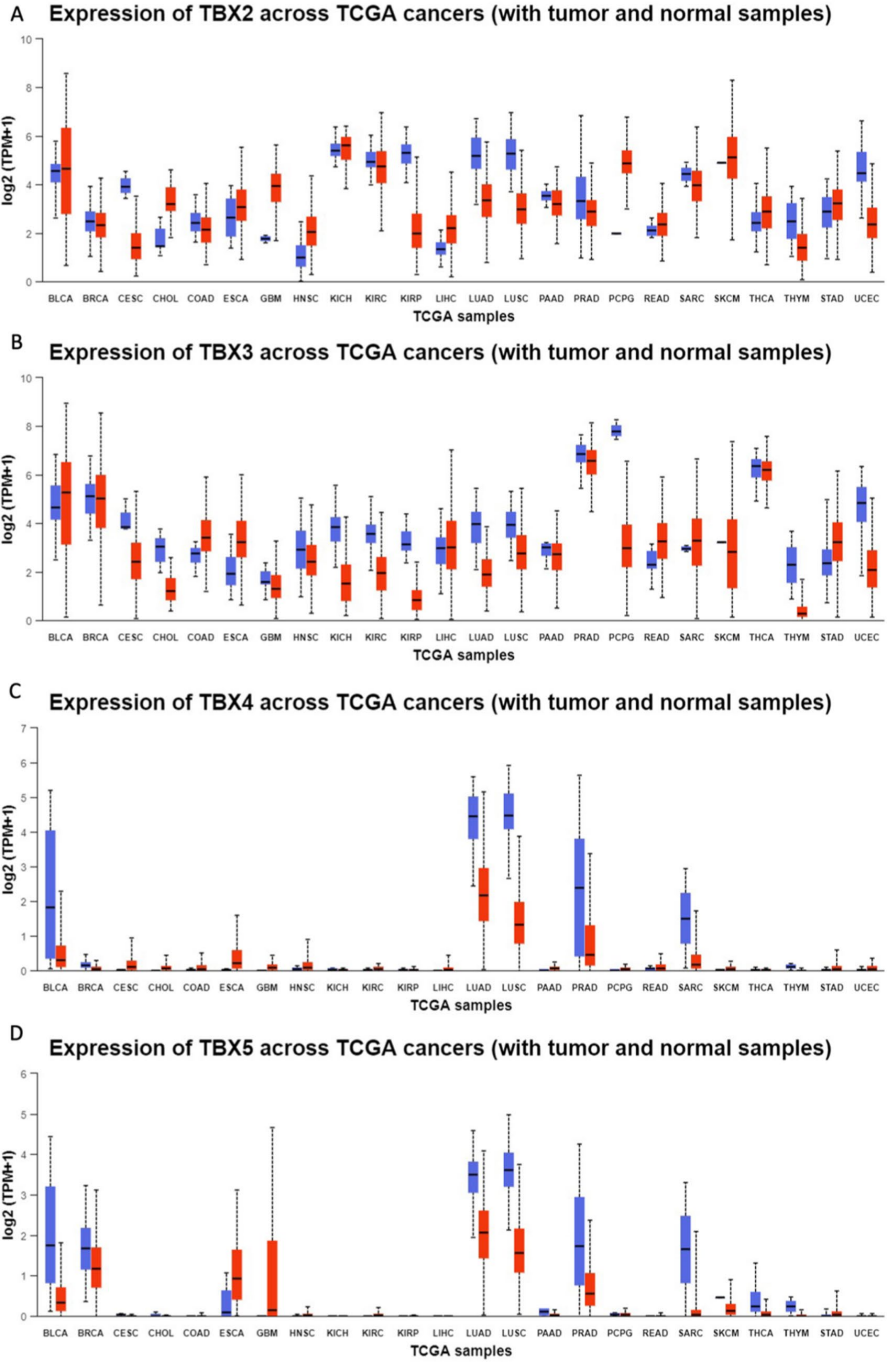
Comparison of TBX2 subfamily expression between tumor and normal samples. Differential expression of **A** *TBX2*, **B** *TBX3*, **C** *TBX4*, **D **TBX5

**Fig. 3:**
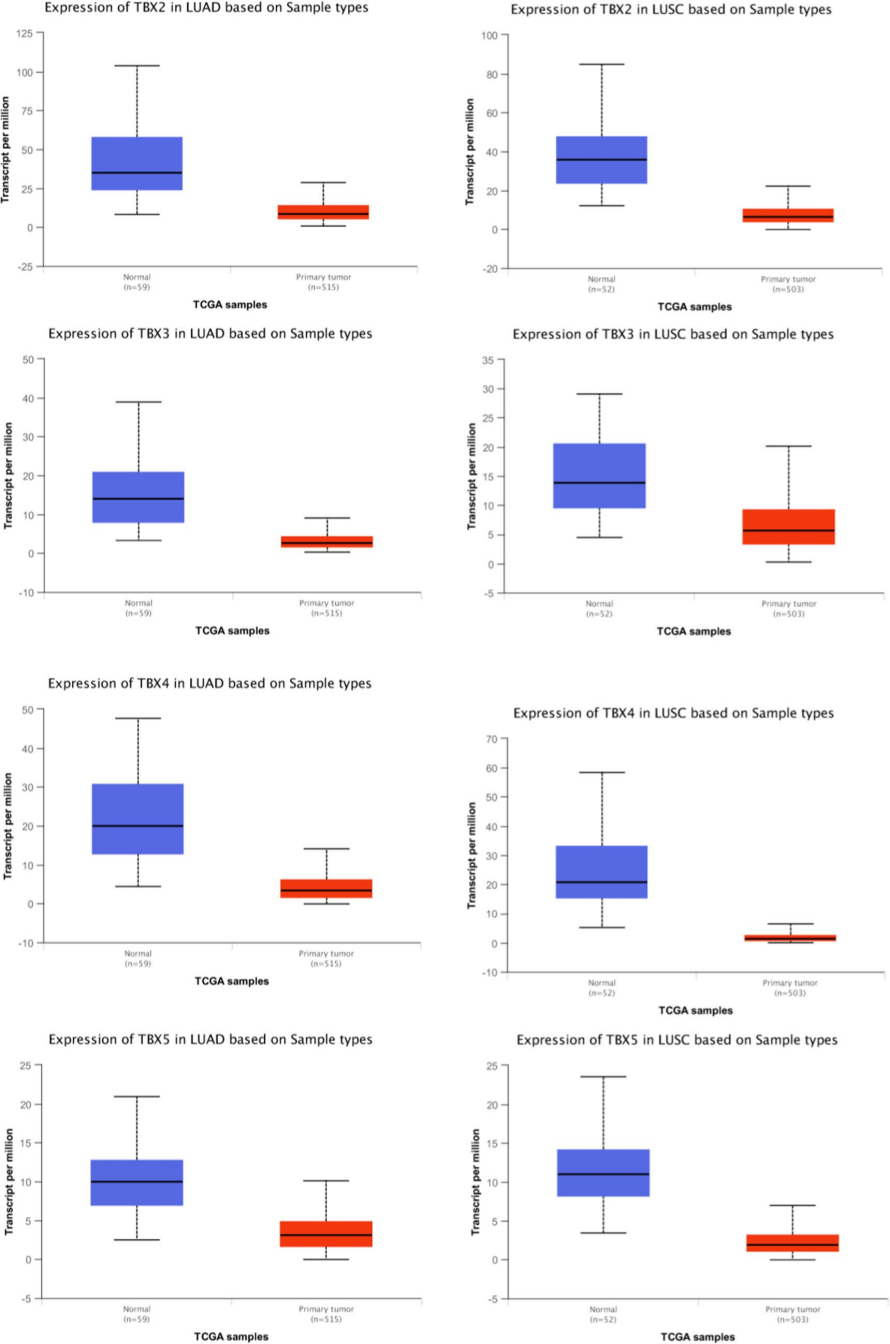
*TBX2* subfamily members were downregulated in LUAD and LUSC (UALCAN). The mRNA expressions of *TBX2* subfamily members were found to be low-expressed in primary LUAD and LUSC tissues compared to normal samples. *p < 0.05

As four members of *TBX2* subfamily of T-BOX transcription factor genes, *TBX2*, *TBX3*, *TBX4* and *TBX5* play important role in various aspects in embryonic development [16]. In addition, *TBX2* and *TBX3* have been reported to be overexpressed in certain cancer types, including endometrial, cervical, ovarian, breast carcinoma [17]. In our study, We compared TBX2 subfamily expression levels between cancer and matched normal samples from 24 cancers, based on UALCAN database (Fig. 2). We found that TBX2 subfamily has differential expression in Bladder Cancer (BLCA), Lung Adenocarcinoma (LUAD), Lung Squamous Cell Carcinoma (LUSC), Prostate Cancer (PRAD) and Sarcoma (SARC) compared to the matched normal tissues. It should be noted that, in both squamous cell and adenocarcinoma of the lung, the expression of TBX2 subfamily genes is significantly reduced in comparison to matched normal tissues (Fig. 3). Figure 3 illustrates that TBX2 is reduced by threefold in lung adenocarcinoma and fourfold in lung squamous cell carcinoma, compared to normal lung tissue. The expression of TBX3 is decreased by fivefold in lung adenocarcinoma and is twofold reduced in lung squamous cell carcinoma. Similarly, TBX4 has been reduced by fourfold and tenfold in lung adenocarcinoma and squamous cell carcinoma, respectively. Lastly, TBX5 was reduced by 2.5-fold in lung adenocarcinoma, and by fourfold in lung squamous cell carcinoma.

### Low expression of TBX2, TBX3, TBX4 and TBX5 was validated in lung squamous cell carcinoma tissues

**Fig. 4:**
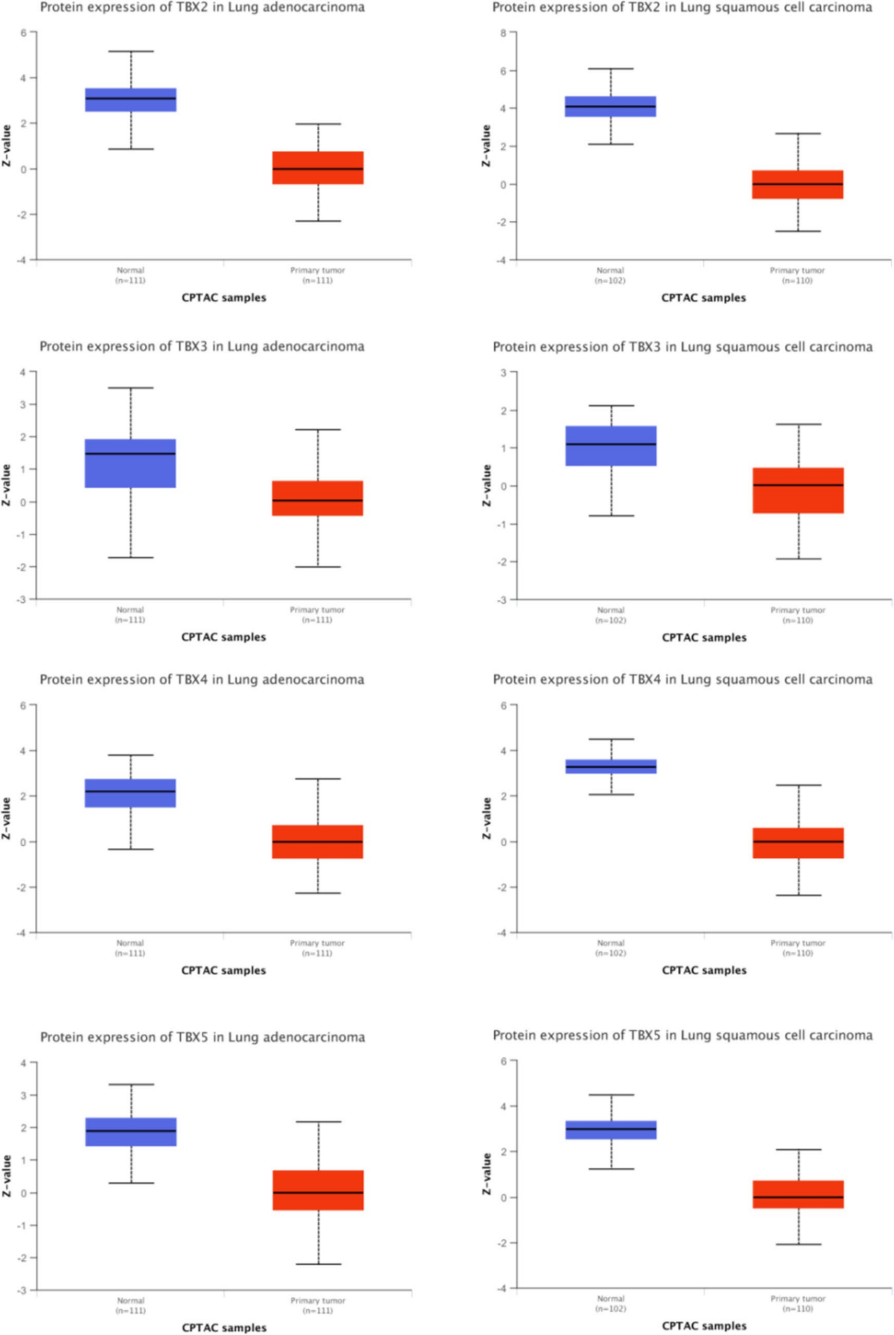
*TBX2* subfamily members protein level were downregulated in LUAD and LUSC (UALCAN). The protein expressions of *TBX2* subfamily members were found to be low-expressed in primary LUAD and LUSC tissues compared to normal samples. *p < 0.05

**Fig. 5:**
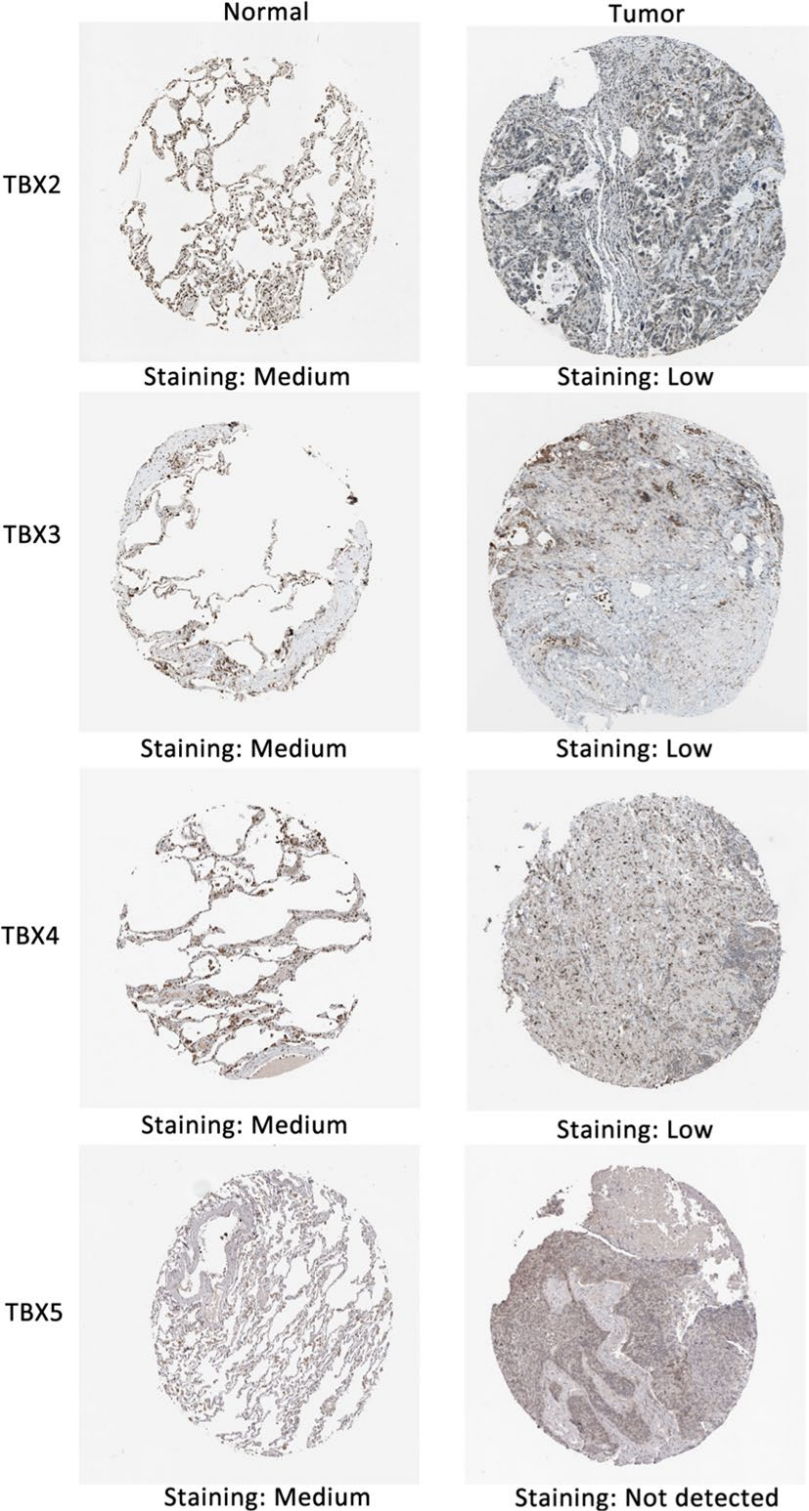
Immunohistochemistry images in normal (N) and tumor tissues (T)

**Fig. 6:**
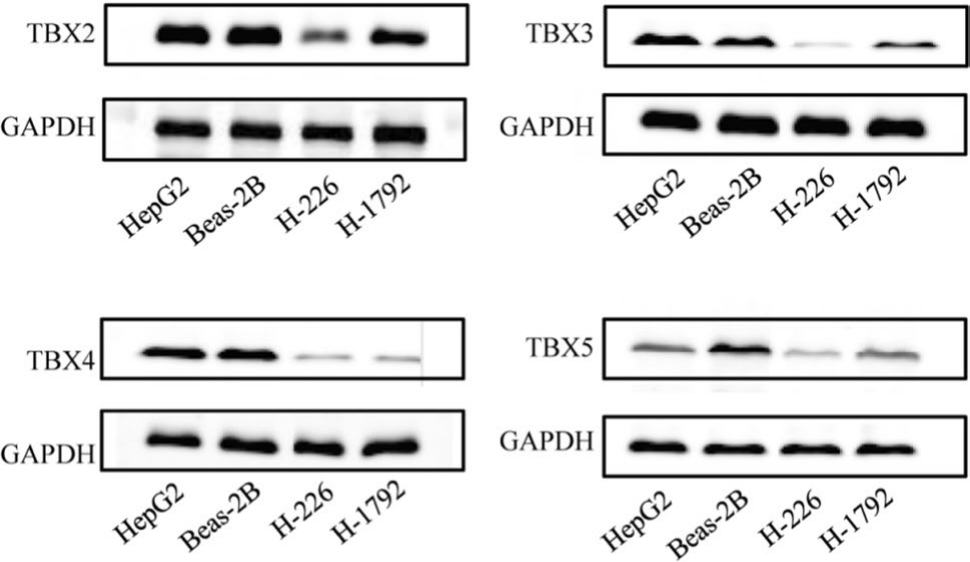
The expression of *TBX2* subfamily in lung cancer cells and normal lung cells. TBX2 subfamily members were downregulated in H-226 and H-1792 lung cancer cell lines. *TBX2* subfamily members were high expression in HepG2 liver cancer cell and Beas-2B normal lung cell

We used the protein expression database of UALCAN to search the protein expression of the TBX2 subfamily (Fig. 4). The protein expression of TBX2 is reduced threefold in pulmonary adenocarcinoma, and fourfold in pulmonary squamous cell carcinoma. Like TBX2, TBX3 has decreased protein expression by 1.5-fold in pulmonary adenocarcinoma, and by onefold in non-squamous cell carcinoma. TBX4 has decreased protein expression by two-fold in pulmonary adenocarcinoma, and by three-fold in pulmonary squamous cell carcinoma. Finally, the protein expression of TBX5 decreased by twofold and 2.5-fold in lung adenocarcinoma and lung squamous cell carcinoma.The result showed that the TBX2 subfamily had low expression in both lung squamous cell carcinoma and lung adenocarcinoma. Then we used Human Protein Atlas to determine the expression of *TBX2*, *TBX3*, *TBX4* and *TBX5* in lung squamous cell carcinoma tissues (Fig. 5). As expected, results showed a significant decrease of *TBX2*, *TBX3*, *TBX4* and *TBX5* expression in lung squamous cell carcinoma tissues compared to paired normal lung tissues, which further supported our hypothesis (Fig. 5).The proteins of TBX2,TBX3,TBX4,TBX5 were medium expressed in lung squamous cell carcinoma tissues, whereas not or low expressions of them were found in normal lung tissues. To further confirm our bioinformatics analysis above, we conducted a protein electrophoresis analysis on lung cancer cell lines, normal lung epithelial cell line, and a control group of liver cancer cell lines (Fig. 6). The results showed that the expression of the TBX2 subfamily gene was downregulated in the H-226 and H-1792 lung cancer cell lines compared to the Beas-2B normal lung epithelial cell line. HepG2, which was used as the control liver cancer cell line, demonstrated stable expression of the TBX2 subfamily gene, but with higher expression levels compared to the lung cancer cell lines.

### Association between mRNA levels of TBX2 subfamily members and prognosis of patients with lung cancer

**Fig. 7:**
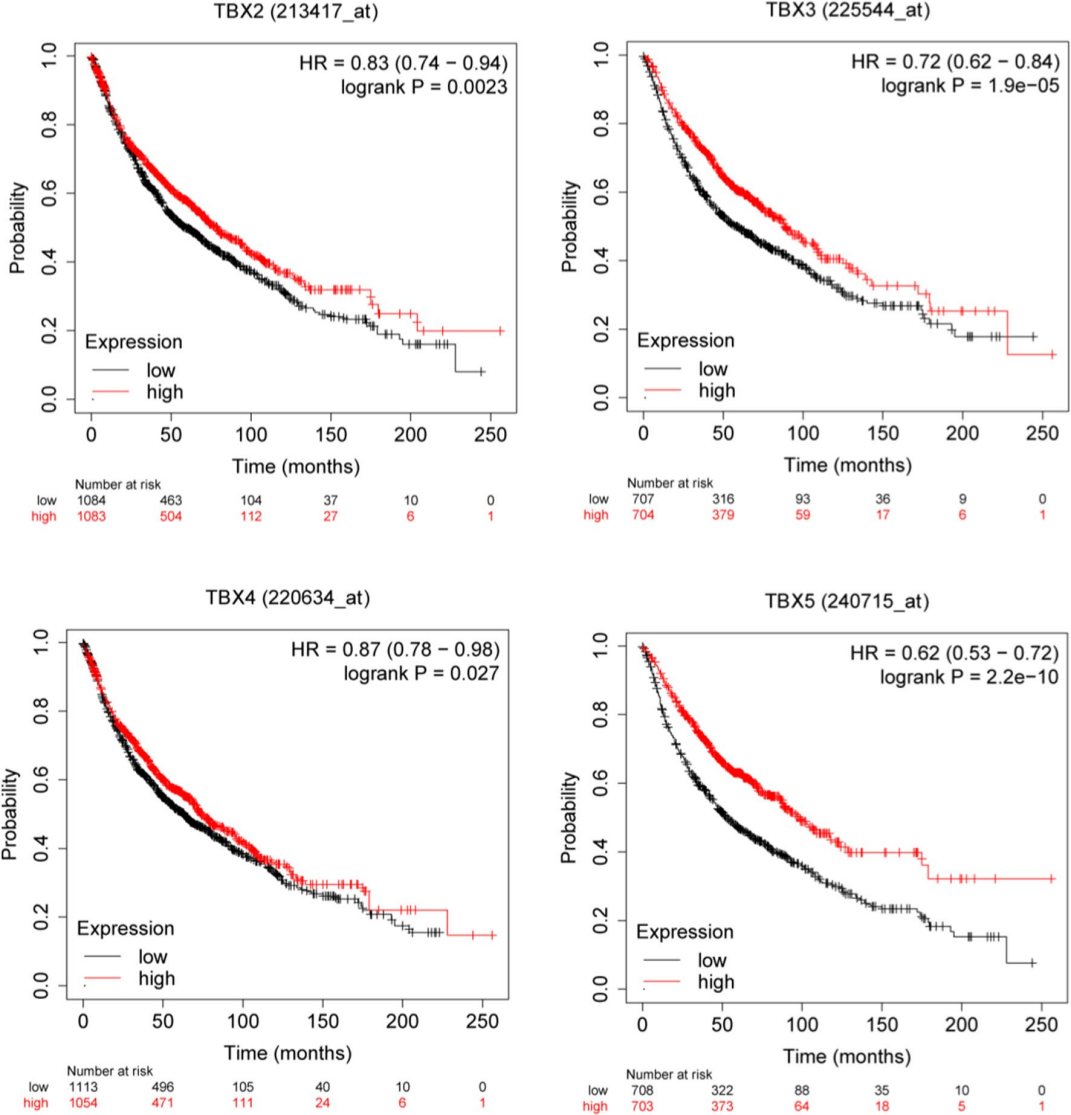
Prognostic value of mRNA expression of distinct *TBX2* subfamily members in lung cancer patients (Kaplan–Meier Plotter)

To assess the effect of *TBX2* subfamily members on non-small cell carcinoma prognosis, Kaplan–Meier analysis was further used in this study (Fig. 7). Herein, we used online Kaplan–Meier plotter to assess the relationship between *TBX2* subfamily expression in 2435 cases of non-small cell carcinoma and their survival. The Kaplan–Meier curve and log-rank test analysis suggested that subfamily members of *TBX2* are all correlated with overall survival (OS) of lung cancer patients (logrank P < 0.05). Moreover, the lung cancer-specific OS rate significantly increased in patients with *TBX2* subfamily high expression group than that in low expression group *(TBX2* logrank P = 0.0023, *TBX3* logrank P = 1.9e-5, *TBX4* logrank P = 0.027, *TBX5* logrank P = 2.2e−10).

### The methylation status of the TBX2 subfamily gene promoter region in human NSCLC

**Fig. 8:**
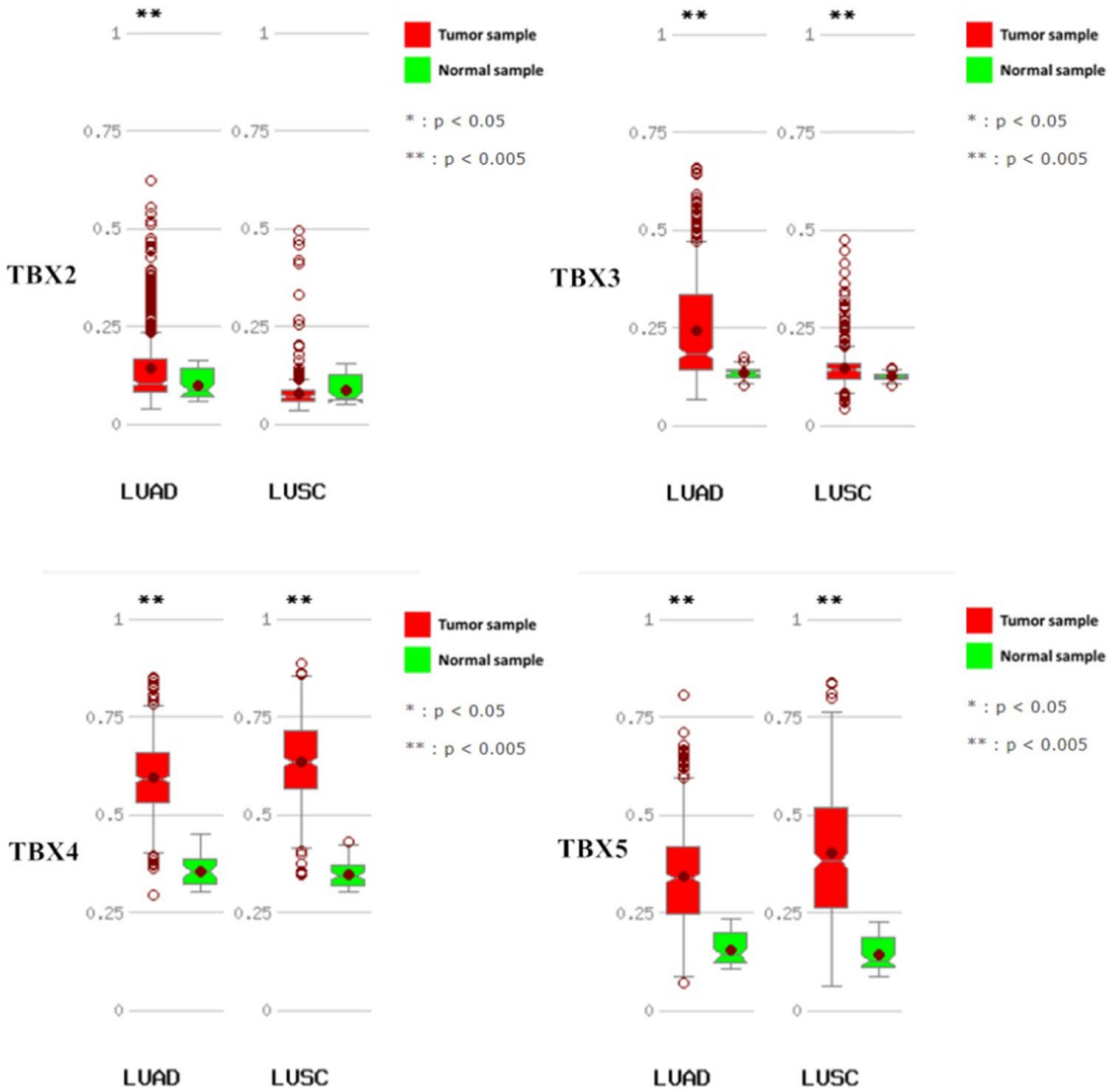
The methylation status of the *TBX2* subfamily gene promoter region in LUAD and LUSC compared to normal lung tissues(MethHC). With the exception of *TBX2* in LUSC, *TBX2* subfamily members' promoter region methylation status are all increased in LUAD and LUSC compared with normal lung tissues. **p < 0.01

**Fig. 9:**
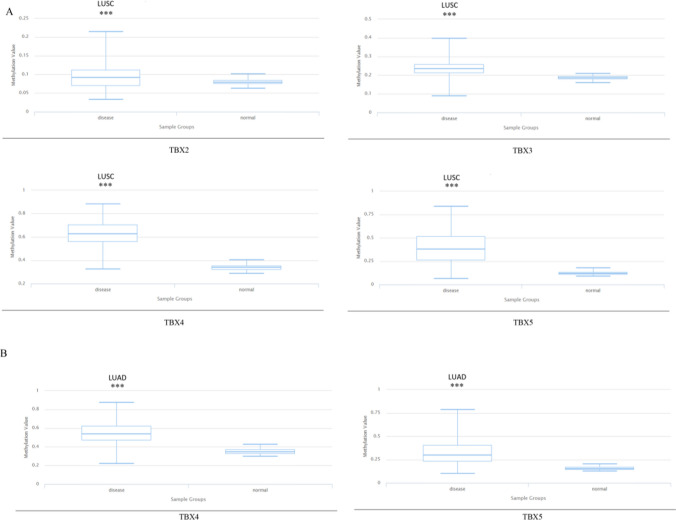
The methylation status of the *TBX2* subfamily gene promoter region in LUAD and LUSC compared to normal lung tissues. (DiseaseMeth version 2.0). **A**. *TBX2*, *TBX3*,*TBX4* and *TBX5*’s promoter region methylation status are all increased in LUSC. **B**. TBX4 and TBX5’s promoter region methylation status are increased in LUAD compared with normal lung tissues. ***P < 0.001

Based on MethHC database, we compared the methylation status of *TBX2* subfamily gene promoter region in human NSCLC and normal lung tissue. The MethHC database's data comes from 471 LUAD patients and LUSC patients. Results showed that *TBX2* subfamily member promoter regions are highly methylated in NSCLC, along with TBX2 in LUSC (Fig. 8). In order to verify the accuracy of the results, we used DiseaseMeth version 2.0 to detect the methylation of *TBX2* subfamily promoter regions (Table 1). *TBX2* subfamily members were highly methylated in NSCLC except *TBX2*, *TBX3* in LUAD (Fig. 9). Based on the above results, we found that TBX2 subfamily member promoter regions were highly methylated in NSCLC.

**Table 1:** The significant changes of TBX2 subfamily member's promoter region methylation status in LUAD and LUSC (DiseaseMeth version 2.0)

Disease name	Genomic region	Transcript	Gene	P-value	meanMethylDisea-meanMethylNormal
LUSC	chr17:59475256–59477756	NM_005994	TBX2	5.19E−09	0.018
LUSC	chr12:115121469–115123969	NM_005996	TBX3	1.62E−12	0.052
LUSC	chr17:59531848–59534348	NM_018488	TBX4	0.00E + 00	0.291
LUSC	chr12:114845747–114848247	NM_080717	TBX5	1.62E−12	0.282
LUAD	chr17:59531848–59534348	NM_018488	TBX4	1.62E−12	0.191
LUAD	chr12:114845747–114848247	NM_080717	TBX5	1.62E−12	0.165

## Discussion

In previous researches, *TBX2* subfamily of transcription factors disorder has been reported to be involved in various human diseases [18]. Until recently, the role of *TBX2* subfamily in the process of carcinogenesis comes into the eyesight of researchers. Although there have been certain reports which clarify the contributions of *TBX2* subfamily members in cancer pathogenesis, the effect of *TBX2* subfamily in carcinogenesis and prognosis of lung cancer still remains elusive. We report for the first time that low expression of Tbx2 subfamily predicts poor prognosis in patients with lung cancer, and further verify the low expression of Tbx2 in pathological specimen of lung cancer. The low expression of *TBX2* subfamily is a poor prognostic factor for NSCLC, which may be related to the increased methylation of promoter region in NSCLC patients. This study is promising to shed light on discovering a reliable prognostic marker for patients after lung cancer resection.

It has been reported that *TBX2*, member of Tbx2 subfamily of transcription factors, was overexpressed in breast and bladder cancer [19, 20]. *TBX2* started mechanisms of normal growth control by recruiting a series of inhibitory compounds to combine with reactive promoter of *EGR1*, which led to uncontrolled proliferation of breast cancer cells [21]. Nevertheless, our research suggested that the expression of *TBX2* was significantly downregulated in lung cancer, which was supported by Khalil’s study [22]. It is worth noting that *TBX2* represented a higher expression in normal lung tissues compared with other specific normal tissues, which suggesting an important role of *TBX2* in embryonic development and morphogenesis of lung [15].

There were conflicting reports about the role of *TBX3* in different types of cancer. On the one hand, it was reported that *TBX3* was overexpressed in most human malignancies such as melanoma, breast, ovarian and bladder cancer [23, 24]. In these studies, the overexpression always predicted metastasis and poor prognosis in patients, which suggested a promoting effect of *TBX3* on malignancy progression in cancer. Study on the mechanism has revealed that *TBX3* promoted invasion of tumors mainly through inhibition of *E-cadherin* expression [25]. On the other hand, our data suggested that the expression of *TBX3* in lung cancer was significantly downregulated, which was contrary to Wu’s study [26]. Besides, *TBX3* correlated with survival of lung cancer patients. Low expression of *TBX3* in lung cancer predicted low survival and poor prognosis of patients, which indicated that *TBX3* was an independent prognosis factor and a potential therapeutic target for OS in lung cancer. *TBX3* might play an inhibitory role in malignancy progression of lung cancer.

A proteomics study revealed the relationship between *TBX4* and malignancy progression in cancer for the first time. This study found that *TBX4* was overexpressed in pancreatic ductal adenocarcinoma tissue (DAC). Thereafter, further research of Meijuan Zong found that *TBX4* was an independent prognostic factor for OS in stage II pancreatic ductal carcinoma (PDC). Low expression of *TBX4* predicted poor prognosis of stage II PDC [14]. High-throughput sequencing technology analysis in lung fibroblasts suggested that *TBX4* in lung cancer associated fibroblasts (CAFs) was downregulated and highly methylated [27]. It indicated that epigenetic silence of *TBX4* was involved in phenotypic alteration of CAFs from lung cancer. Similar to the result of Horie M, our data showed that the expression of *TBX4* in lung cancer tissues showed a significant decrease compared to normal tissues. Besides, low expression of *TBX4* predicted poor prognosis of lung cancer patients. *TBX4* was associated with tumor stage of lung cancer, similar to the result in PDC.

Earlier studies had shown that *TBX5* played an important role in cardiac and forelimb development in mammals [28]. However, the role of *TBX5* in malignancy progression in cancer still remains elusive. Rosenbluh reported that in the development of colon cancer, the compounds formed by *β-catenin*, yes-associated protein 1 (*YAP1*) and *TBX5* were essential for survival of colon cancer, similar phenomena were observed in other tumors [29]. High expression of *TBX5* had been verified to be related with low survival rate of stage I and II gastric cancer patients [30, 31]. However, our research finding proved that the expression of *TBX5* was downregulated in lung cancer. Surprisingly, low expression of *TBX5* turned out to be associated with a decrease in OS, which predicted a poor prognosis in lung cancer. In conclusion, our research finds that the expression of all four *TBX2* subfamily members is downregulated in lung cancer tissues.

In our research, the TBX2 subfamily gene shows low expression in non-small cell lung cancer. Through survival analysis, we found that the low expression of the TBX2 subfamily gene in non-small cell lung cancer patients indicates poor prognosis. Finally, through mutual verification analysis of two methylation bioinformatics databases, it is suggested that the low expression of the TBX2 subfamily in non-small cell lung cancer patients may be highly correlated with methylation in its promoter region. Our study is promising to shed light on discovering a novel reliable cancer marker for prognosis of lung cancer patients.

## Materials and methods

### Ethics statement

This study was approved by the Academic Committee of Wuxi 9Th People's Hospital Affiliated to Soochow University and conducted according to the ethical standards formulated in the Helsinki Declaration. It was confirmed that all written informed consents were obtained as all data sets were retrieved from published literatures.

### Classification and gene structure of TBX2 subfamily

The HUGO Gene Nomenclature Committee (HGNC) assigns unique symbols and names to human genes based on the European Bioinformatics Institute (EMBL-EBI). In current, the HGNC database not only integrates more than 40000 approved gene symbols, but also curates genes into family sets based on shared characteristics such as homology, function or phenotype. Herein, we retrieved basic information of Tbx2 subfamily from the HGNC database (http://www.genenames.org/cgi-bin/genefamilies/set/766) [32]. *TBX2* subfamily contains four members, including *TBX2*, *TBX3*, *TBX4* and *TBX5*. Then, we retrieved detailed information about TBX2 subfamily through the search terms of *TBX2*/homo, *TBX3*/homo, *TBX4*/homo and *TBX5*/homo from the NCBI (National Center for Biotechnology Information) database. Finally, graphic mode of structure of TBX2 subfamily transcription products including coding region, untranslated coding region (UTR) and T-BOX domain was drawn with software.

### UALCAN analysis

UALCAN gene expression array datasets is a cancer microarray database and online data-mining platform which aimed at facilitating discovery from genome-wide expression analysis [33]. Herein, we use this database to analyze the transcriptional level of TBX2 subfamily genes in different types of cancers. We compared the expression level of TBX2 subfamily in clinical cancer specimens with that in normal controls and used Student’s t test for result analysis. The cutoff of P value and fold change were defined as 0.01 and 1.5, respectively. Gene rank: 10%, data type: mRNA. We also performed protein expression analysis of the TBX2 subfamily in lung squamous cell carcinoma and lung adenocarcinoma compared to normal lung tissue using the protein expression module of the UALCAN database.

### Immunohistochemistry (IHC) staining

To evaluate differences in TBX2 subfamily expression at the protein level, IHC images of TBX2 subfamily protein expression in normal tissues and Lung Squamous Cell Carcinoma were downloaded from the The Human Protein Atlas (http://www.proteinatlas.org/) and analyzed [34]. The Human Protein Atlas serves as a comprehensive database housing information pertaining to the proteome of the human body, encompassing details regarding their distribution, expression levels, and functional roles within diverse tissues and cells. This resource furnishes detailed imagery, gene expression profiles, and other pertinent data, facilitating the comprehension of the pivotal roles played by proteins in the context of both health and disease.

### Cell culture

H-226, H-1792, Beas-2B, and HepG2 cell lines are gifts donated by the Clinical Medical College of Suzhou University. All the above-mentioned cells were cultured in an environment with a temperature of 37 ℃ and a gas environment of 5% CO2. All cell lines were cultured in Dulbecco's modified Eagle's medium (DMEM) containing 10% fetal bovine serum and 1% penicillin–streptomycin mixture (HyClone, USA). After being resuscitated from liquid nitrogen, each cell line was passaged 15 times before being used for subsequent experiments. Cell culture was performed using a 6-well plate with a cell density close to 80%, approximately 5*10^5 cells were used for protein extraction.

### Western blotting analysis

To prepare protein lysate, cells were lysed with RIPA lysis buffer supplemented with protease inhibitors to reduce protein degradation. The lysate was maintained at an icy temperature during the entire protein extraction process to uphold sample integrity. The proteins were then extracted by centrifugation of the lysate at 1400 g for 15 min under 4 ℃. The protein concentration of the extracted proteins was quantified with the BCA protein assay kit. After protein extraction, SDS-PAGE gel electrophoresis was performed to separate the protein, which was then transferred onto a PVDF membrane. The PVDF membrane was shaken in 5% skim milk, and then blocked overnight at 4 ℃ in a wet box containing primary antibodies against TBX2(Abcam ab33298), TBX3(Abcam ab99302), TBX4(Santa Cruz sc-515196), TBX5 (Abcam ab137833)and GAPDH(Santa Cruz sc-47724).On the following day, after 1 h of incubation at room temperature with secondary antibodies, analysis was conducted using an ECL protein imprint imaging device. The dilution ratio for the secondary antibody is 1:2000.

### Kaplan–Meier plotter

The Kaplan–Meier plotter (http://kmplot.com/analysis/) enables to assess the effect of any gene or gene combination on survival in various types of tumors using over 50,000 samples measured with gene arrays, RNA-seq or next generation sequencing [35]. The Kaplan–Meier plotter (K-M plotter) is capable of assessing the effect of up to 54 k genes on survival of 21 cancer types and the largest datasets contain breast (n = 6,234), lung (n = 3,452), ovarian (n = 2,190), and gastric (n = 1,440) cancer. Herein, we used the K-M plotter to evaluate the prognostic value of mRNA expression of distinct *TBX2* subfamily genes in lung cancers. In K-M plotter, we divided cancer participants into low and high expression group according to median values of *TBX2* subfamily mRNA expression. The prognostic value of *TBX2* expression was validated by K-M survival curves. Information about the number-at-risk cases, median values of mRNA expression levels, HRs, 95% CIs and p-values is all available on the K-M plotter website. P < 0.05 was considered statically significant.

### DNA methylation database

MethHC was a database for human cancer gene expression and methylation [36]. The correlation between TBX2 subfamily expression and its methylation were confirmed by the Pearson correlation coefficients. P < 0.05 has statistical significance. DiseaseMeth 2.0 is a database that aims to provide the most complete collection and annotation of abnormal DNA methylation in human diseases, especially in various cancers [37]. The database includes 175 large-scale methylation datasets of 88 diseases and more than 14000 accurate experimental information extracted from PubMed. The DNA methylation index is calculated based on the mean methylated intensity (M) and unmethylated intensity (U) at each locus in every sample, using the formula (β = M/ [M + U]), and is presented as β values.

### Statistical analysis

The Student's t-test was employed to identify variations in the expression of TBX2 subfamily mRNA between cancerous tissues and adjacent normal lung tissues. We utilized the log-rank test to evaluate the potential correlation between the expression of TBX2 subfamily proteins and the overall survival (OS) of lung cancer patients. A p < 0.05 is deemed to be statistically significant.

## Data Availability

The data that support the findings of this study are available from the corresponding author upon reasonable request. The data used and/or analyzed during the current study are available from the corresponding author on reasonable request.
